# Research on the influence mechanism of health anxiety on hypochondriasis in older adults from the perspective of digital health literacy theory: mediated by information search

**DOI:** 10.3389/fpubh.2025.1672145

**Published:** 2025-10-08

**Authors:** Aojie Chen, Xiquan Wang

**Affiliations:** Nanjing University of Chinese Medicine, Nanjing, China

**Keywords:** health information search, health anxiety, geriatric hypochondriasis, digital health literacy, mediation analysis

## Abstract

**Objective:**

This study aims to elucidate the correlations among health information search, health anxiety, and geriatric hypochondriasis, and to examine the mediating role of health information search behavior between health anxiety and hypochondriasis among the older adults, thereby providing a theoretical basis for interventions.

**Methods:**

A cross-sectional survey was conducted among 251 older adults participants recruited via cluster sampling from six streets in Changshu City, Suzhou, from January to March 2024. Data were collected using validated scales, including the Short-Form Health Anxiety Scale and the Short-Form Cyberchondria Severity Scale. SPSS 26.0 was used for statistical analysis, incorporating descriptive statistics, correlation analysis, binary logistic regression, and bootstrap mediation analysis (5,000 samples). Statistical significance was set at *p* < 0.05.

**Results:**

(1) More than 60% of the participants were female; 44.22% were aged 60–65; 46.22% self-rated as healthy; 41.43% frequently searched for health information. (2) Health information search and health anxiety were positively correlated with geriatric hypochondriasis (both *p* < 0.01). (3) Health information search fully mediated the relationship between health anxiety and hypochondriasis (mediating effect = 0.659, 95% CI [0.41, 0.92]).

**Conclusion:**

This study confirms the mediating role of health information search in the pathway from health anxiety to hypochondriasis among the older adults. It suggests that interventions should focus on improving digital health literacy and reducing unnecessary health information searches to mitigate hypochondriacal tendencies.

## 1 Introduction

### 1.1 Background

As the Internet trend swept the world, the health anxiety of the older adults population experiencing COVID-19 increased.79% of Chinese smartphone users prefer to search for health information via mobile, to a wide audience. While health information search is a fast, convenient, and low-cost way to obtain health information. However, due to the uneven quality of information, older adults users are likely to be unable to effectively judge the authenticity due to the relative lack of information quality, so they mistakenly believe in untested online health information and use it as an effective tool for self-diagnosis, and contradictory health information will make them search for health information excessively to verify the authenticity, even if they go to medical institutions for treatment, they still suspect their own health problems. In the end, it will cause damage to one’s own health and personal economy. At a time when Internet penetration continues to increase, there are few studies on the influencing mechanism of hypochondriasis and health anxiety in geriatrics, especially on the relationship between health anxiety and hypochondriasis. Therefore, it is necessary to take the older adults as the research object, to gain an in-depth understanding of the impact of health anxiety on geriatric hypochondriasis in China from the perspective of public health management, to promote the understanding and attention to geriatric hypochondriasis, and to implement the “Healthy China” strategy in the context of aging.

### 1.2 Purpose of the study

This study investigates the current status of health anxiety and hypochondriasis among the older adults and explores the mediating role of health information search. The findings may inform better health services and provide a reference for future research.

### 1.3 Research implications

#### 1.3.1 Theoretical implications

At present, many literatures have analyzed the impact of health anxiety on health information search, the mechanism of health information search in the population, and the sources and influencing factors of information demand, but few studies have studied the relationship between health anxiety and geriatric hypochondriasis. In recent years, a large number of older adults patients with health anxiety blindly believe in online health information, their compliance with doctors has declined, and finally they have lost both money and money, so this study hopes to further explore the impact of health anxiety on geriatric hypochondriasis and provide a reference for further research on the logical framework between the two in the future.

#### 1.3.2 Practical significance

China has long entered the “aging,” and the older adults population is the main force in the use of the health system. By understanding the health information on the Internet, the older adults can reduce the number of medical visits, reduce the medical expenses of the older adults to a certain extent, improve the work efficiency of medical institutions, and reduce the pressure caused by the uneven distribution of medical resources. However, as the older adults’s fear of possible illness has an increasingly significant impact on their behavior in seeking medical services, especially in the current situation of inconvenient medical treatment, the use of online services such as “Internet + medical care” has become particularly critical. However, due to the degenerative changes in body and mind, the older adults lack the ability to screen, process and use health information to a certain extent, and are prone to excessive health information search, thus posing a threat to their own life safety. The results of this study are helpful to clarify the impact of health anxiety on hypochondriasis in the older adults and put forward relevant suggestions to guide them to understand health information, guide them to seek medical treatment reasonably, and solve health problems.

### 1.4 Concept identification

#### 1.4.1 Health anxiety

Theoretical definition: health anxiety refers to the psychological reaction due to external environmental stimuli, which is manifested as excessive attention to a certain physical symptom or excessive fear and worry about the possibility of suffering from a serious disease. We have a normal level of concern about our health, but this can sometimes turn into a persistent, excessive fear of serious illness, often referred to as health anxiety.

Operation definition: clinical anxiety scales such as the State–Trait Anxiety Scale and the Simplified Health Anxiety Scale were used to measure the clinical anxiety disorder scale.

#### 1.4.2 Health information search

Theoretical definition: health information search is the behavior of people in a certain situation to meet their health needs, purposefully search for all relevant information related to health, risk, disease and health protection through online tools.

Operational definition: for the sake of quantification, this study measures health information search in a quantitative dimension.

#### 1.4.3 Geriatric hypochondriasis

Theoretical definition: geriatric hypochondriasis Geriatric hypochondriasis refers to the excessive and repeated search for personal health information by older people online, causing them to feel frustrated, anxious, and even unable to control their search behavior.

Cognitive-emotional factors, such as health concerns, and behavioral factors, such as searching for medical information too frequently, can be considered the most prominent features of hypochondriasis in old age.

**Figure 1:**
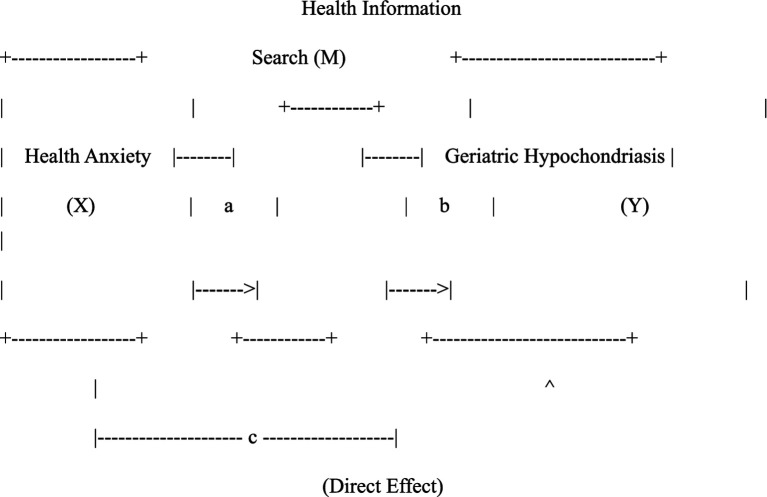
The hypothesized mediation model of health information search on the relationship between health anxiety and geriatric hypochondriasis. X = independent variable; M = mediator; Y = dependent variable. Path *a* represents the effect of health anxiety on health information search. Path *b* represents the effect of health information search on geriatric hypochondriasis controlling for health anxiety. Path c’ represents the direct effect of health anxiety on geriatric hypochondriasis controlling for the mediator. The total effect of X on Y is denoted as *c* (where c = c’ + ab*). The bootstrap results indicated that health information search fully mediated the relationship (mediating effect = 0.659, 95% CI [0.41, 0.92]).

Operational definition: In this study, the Short Hypochondriacal Scale, also known as the Chinese version of the Geriatric Hypochondriacal Severity Scale, was used. To assess the validity, reliability, and applicability of the severity of excessive health information searches on the Internet in China. The scale exhibits high internal consistency, as well as sufficient concurrency and convergence validity, and the criterion-related validity of the scale is supported by a significant correlation between geriatric hypochondriasis and health anxiety and compulsion (Figure 1).

## 2 Research design and methods

### 2.1 Research subjects

Changshu City, Jiangsu Province. Inclusion criteria were: (1) age ≥60 years; (2) voluntary participation with informed consent.

### 2.2 Sampling method

Cluster sampling was used. Fifty questionnaires were distributed in each of the six streets, totaling 300. After excluding invalid responses, 251 questionnaires were retained (effective response rate: 83.67%).

### 2.3 Measures

All scales were previously validated in Chinese populations.

General Information Questionnaire: Collected demographics, self-rated health, and health information search frequency.

Short-Form Health Anxiety Scale (SHAI): 8 items, total score 0–24. Score ≥6 indicates health anxiety.

Short-Form Cyberchondria Severity Scale (CSS-12): 12 items rated on a 5-point Likert scale. Higher scores indicate greater hypochondriasis.

### 2.4 Research tools

The questionnaires used in this study included the General Status Questionnaire, the Health (1), the Simplified Health Anxiety Scale (2), and the Simplified Internet Hypochondriacal Inventory (1). In accordance with the Declaration of Helsinki, this study was approved by the Ethics Committee of Nanjing University of Chinese Medicine. All participants provided written informed consent and were informed of the research purpose and data confidentiality. This study received ethical approval from the Institutional Review Board of the School of Health Economics and Management at Nanjing University of Chinese Medicine (Approval Date: December 11, 2023). The research protocol adhered to ethical principles of fairness, non-maleficence, and respect for participant autonomy. All participants were aged 60 years or older and resided in Suzhou, China. Prior to data collection, participants were fully informed about the study’s objectives, procedures, and their right to withdraw at any time without penalty. Informed consent was obtained voluntarily from all participants before they completed the face-to-face questionnaire.

The Institutional Review Board confirmed that the study design posed no harm or risks to participants and ensured the protection of their rights and privacy. Personal data collected during the survey were anonymized to prevent identification, aligning with privacy protection standards. The recruitment process strictly followed principles of voluntary participation and transparency, with no conflicts of interest or violations of legal or ethical norms identified. All procedures complied with institutional ethical guidelines and national regulations governing human subject research in China.

#### 2.4.1 General situation research

Through the literature survey method and under the guidance of the teacher, a total of 4 questions were investigated for the general situation of the older adults group. Includes:

1. Demographic characteristics: gender, age.
2. Personal health status: self-rated health.
3. Frequency of access to health information.

#### 2.4.2 Short-form health anxiety scale (2)

The total score is 0–54, and 15 is the threshold for screening for health anxiety. In other literatures, similar questions were combined to make a total of 8 questions, with a total score of 0–24. 6 points is a threshold.

#### 2.4.3 Short-form network hypochondriacal scale (1)

The reliability and validity of the short version of the network hypochondriasis scale have been verified in Chinese samples. Consists of 12 entries and is scored using a 5-point score, with higher scores indicating higher cyber hypochondriasis. In other literature, four dimensions were used to measure it, with a total of 4 questions, which were excessive attention, search anxiety, help-seeking, and negative interference.

### 2.5 Statistical analysis

The questionnaire items and options were coded, and the statistical analysis software SPSS 26.0 was entered for statistical analysis after verification, and the test level *p* < 0.05 was valid.

1. Descriptive statistical methods were used to analyze the general demographic characteristics of the older adults over 60 years old, as well as the current situation of health information search, health anxiety and geriatric hypochondriasis.
2. Spearman correlation analysis was used to clarify the correlation between health search frequency, health anxiety, and geriatric hypochondriasis.
3. Binary Logit regression was used to analyze the positive predictive effects of health anxiety and health information search on hypochondriasis in the older adults.
4. The bootstrap method (sampling replicates 5,000 times) was used to construct a 95% confidence interval to test the significance of the mediating effect (3).

## 3 Research results

### 3.1 Descriptive statistical analysis

#### 3.1.1 General information of the research subject

A total of 251 older adults were included in this study. More than 6 of the gender composition are “female,” and 31.47% are male. In terms of age, the number of people “60–65” is relatively large (44.22%). 46.22% of the older adults reported that their health choices were “healthy.” The frequency of health information search is relatively large, with 41.43% (Table 1).

**Table 1:** General characteristics of participants (*n* = 251).

Variable	Category	Frequency (*n*)	Percentage (%)
Gender	Female	151	60.16
	Male	100	39.84
Age	60–65 years	111	44.22
	66–70 years	67	26.69
	≥71 years	73	29.09
Self-rated health	Healthy	116	46.22
	Fair/Poor	135	53.78
Health information search	Frequent/Very frequent	104	41.43

#### 3.1.2 Participants’ health anxiety scores

According to the indicators, a score greater than 6 points on the scale is associated with some degree of health anxiety disorder. According to the descriptive statistical analysis, the score of the Health Anxiety Scale was 12.689 ± 4.847 points, and the sub-indicators fluctuated between 2.5 ± 0.8. The percentile indicator showed that 90% of the sample population suffered from health anxiety (Table 2).

**Table 2:** Descriptive statistics of health anxiety scale scores (*n* = 251).

Variable	Sample size	Min	Max	Mean (±SD)	Median
Total Score (Short Health Anxiety Scale)	251	0	24	12.69 ± 4.85	3
C1: Perception of Pain Severity	251	1	4	2.55 ± 0.94	3
C2: Control Over Disease-Related Thoughts	251	1	4	2.59 ± 0.89	3
C3: Awareness of Bodily Changes	251	1	4	2.61 ± 0.81	3
C4: Self-Identification with Disease	251	1	4	2.51 ± 0.87	3
C5: Subjective Assessment of Disease Risk	251	1	4	2.62 ± 0.89	3
C6: Family’s Perception of Health Concerns	251	1	4	2.61 ± 0.84	3
C7: Ability to Enjoy Life with Severe Illness	251	1	4	2.66 ± 0.81	3
C8: Confidence in Modern Medicine	251	1	4	2.55 ± 0.90	3

#### 3.1.3 Hypochondriacal scale score of study subjects

There is currently no unified threshold for geriatric hypochondriasis, but the higher the score, the more severe the geriatric hypochondriasis. According to statistics, the scores are basically normally distributed, of which 12–16 points (the total score is 20 points) accounts for nearly 60%. Sub-scores show consistency (Table 3).

**Table 3:** Distribution of hypochondriasis scores (total: 20 points).

Score range	Frequency (*n*)	Percentage (%)
12–16 points	151	60.16
Other ranges	100	39.84

### 3.2 Correlation analysis between variables

Using the Pearson correlation coefficient analysis, it was found that:

In short, there was a significant positive correlation between the pairs (4) (Table 4).

**Table 4:** Correlation analysis between variables (Pearson’s r).

Variable	Health anxiety	Health information search	Geriatric hypochondriasis
Health anxiety	1	0.45**	0.52**
Health information search	—	1	0.38**
Geriatric hypochondriasis	—	—	1

**p < 0.01, *p* < 0.01.

### 3.3 Binary logit regression

Firstly, the scores of the Health Anxiety Scale and the Geriatric Hypochondriasis Scale were summed, and according to the clinical criteria, those whose scores exceeded the critical value were determined to have a high level of health anxiety and a tendency to geriatric hypochondriasis, and the data were coded as “1,” otherwise the health anxiety level was low and normal (healthy state). After the data were encoded, the frequency of B health information search, C health anxiety were used as independent variables, and D senile hypochondriacal disease was used as the dependent variable for binary Logit regression analysis, and it can be seen from the following table that the model formula is: ln(p/1-p) = −1.476 + 0.541*B health information search frequency + 2.150*C health anxiety (where p represents the probability that D’s geriatric hypochondriasis is 1, and 1-p represents the probability that D’s senile hypochondriacal disease is 0) (5).

The regression coefficient of the frequency of health information search was 0.541, and showed a significance of 0.05 (*z* = 2.510, *p* = 0.012 < 0.05), and the frequency of health information search had a significant positive impact on geriatric hypochondriasis and an odds ratio (OR value) of 1.717, implying that when the frequency of health information searches increases by one unit, the change (increase) in geriatric hypochondriasis is 1.717 times.

The regression coefficient of health anxiety was 2.150, and it showed a significant level of 0.01 (z = 5.025, *p* = 0.000 < 0.01), which means that health anxiety has a significant positive impact on hypochondriasis in the older adults and an odds ratio (OR value) of 8.585, implying that when health anxiety increased by one unit, the change (increase) in geriatric hypochondriasis was 8.585 times.

The summary analysis shows that the frequency of health information search and health anxiety have a significant positive impact on geriatric hypochondriasis (Table 5).

**Table 5:** Binary logistic regression results (*n* = 251).

Variable	Regression coefficient (β)	Std. error	Odds ratio (OR)	95% CI	*p*-value
Health information search frequency	0.541	0.215	1.717	[1.12, 2.63]	0.012*
Health anxiety	2.150	0.428	8.585	[3.71, 19.85]	0.000***

*p < 0.05, *p* < 0.05, ***p < 0.001, *p* < 0.001.

Through the analysis, the overall prediction accuracy of the research model was 87.00% (>80%), and the model fit was good.

### 3.4 Mediator effect test

First, standardize the coding of variables that incorporate the mediation model. Then, Logit regression was performed to determine the mediating role of health information search behavior in the relationship between health anxiety and geriatric hypochondriasis (6). According to the analysis, there are three models involved in the mediating effect analysis, which are as follows:

Geriatric Hypochondriacal Scale score (0–1) = 4.544–0.451 * Your gender + 0.074 * Your age group: −1.044 * self-rated health + 2.027 * Short Form Health Anxiety Scale score (0–1).
Frequency of health information search = 4.125 + 0.010 * Your gender - 0.088 * Your age group: −0.348 * self-rated health + 0.433 * Short Form Health Anxiety Scale score (0–1).
Geriatric Hypochondriacal Scale score (0–1) = −1.734-0.467 * Your gender + 0.208 * Your age group: −0.514 * self-rated health + 1.368 * Short Health Anxiety Scale score (0–1) + 1.522 * Frequency of health information searches.

The results showed that the total effect was 2.027, the mediating effect of A*B was 0.659, and the direct effect of A*C was 1.368. In line with research hypothesis H4 (Table 6).

**Table 6:** Mediation effect test (bootstrap method).

Path	Effect size	Std. error	95% CI
Total effect (Health Anxiety → Hypochondriasis)	2.027	0.301	[1.45, 2.61]
Direct effect	1.368	0.278	[0.82, 1.91]
Mediation effect (health information search)	0.659	0.129	[0.41, 0.92]

## 4 Discussion

### 4.1 Main findings

#### 4.1.1 Current status of health search frequency among the older adults

In this study, 55% of the older adults are searching for health information frequently or frequently, first of all, because of the rapid development of the Internet, which allows the older adults to easily obtain medical information without leaving home, and some marketing accounts on the Internet often have some eye-catching titles to attract traffic. Once the older adults search for some health information, there will be many related health information pushes, and eye-catching titles will be added to attract the older adults to click, and eventually the older adults will be unable to stop and be trapped in the cocoon of health information.

#### 4.1.2 Current status of health anxiety in the older adults population

In this study, 90% of older adults had some degree of health anxiety symptoms, highlighting the severity of the problem and the urgency to address it. The total score of the overall health anxiety of the study subjects was (12.689 ± 4.847), which may be due to the fact that COVID-19 has affected the health concept of the older adults to a certain extent, and promoted geriatric hypochondriasis. Therefore, the society should pay attention to the electronic health literacy of the older adults, cultivate their correct health concepts, and actively guide the older adults to make rational use of health information to promote their physical and mental health.

#### 4.1.3 Current status of geriatric hypochondriasis

The results of this study showed that the overall total geriatric hypochondriacal score of the study participants was similar to that of Faruk Caner Yam et al. in subjects aged 17 to 65 years through an online platform, suggesting that hypochondriasis has become a social problem. Therefore, geriatric hypochondriasis must be regarded as an important public health issue, focusing on the understanding of geriatric hypochondriasis and health information search behavior from the individual level and social level of the older adults, so as to reduce the adverse impact of geriatric hypochondriasis on the operation of medical institutions.

#### 4.1.4 Health information search, correlation analysis of health anxiety and geriatric hypochondriasis

In this study, there was a positive correlation between health information search and health anxiety among older adults, that is, individuals were more likely to cause health anxiety if they continued to search for health information online (7).

There was a positive correlation between health information retrieval and hypochondriasis in the older adults (8). Failure to understand and use health information, and inability to distinguish the authenticity of information, will promote the occurrence of hypochondriasis in the older adults. This may be related to the fact that older adults with lower levels of e-health literacy have less ability to identify the authenticity and reliability of online health information and make use of it. Therefore, in the process of online health information retrieval, it is not easy to think that you have completed a complete search, so that you cannot rationally stop unnecessary searches. Therefore, it is easy to increase the likelihood of hypochondriasis in old age.

There was a positive correlation between health anxiety and hypochondriasis in the older adults, which was consistent with previous studies. At the mechanism level, although it is not clear whether health anxiety causes geriatric hypochondriasis or geriatric hypochondriasis first and then health anxiety, it can be said that the two promote and depend on each other in their respective occurrence and development processes.

#### 4.1.5 The mediating role of health information search

The study found that health information search behavior was completely mediated. According to cognitive-behavioral theory, older adults with higher levels of health anxiety are more likely to misinterpret vague medical information, health checks, and disease descriptions from the Internet. This misconception may exacerbate health anxiety in older adults and lead to the act of over-searching for health information, known as hypochondriasis. The findings suggest two important points: First, children should provide support to older adults relatives when they have health anxiety. If necessary, you can accompany them to the hospital for treatment and relieve their health anxiety through professional medical diagnosis; Second, online medical consultation platforms need to implement age verification for older adults users and provide more responsible consulting services.

#### 4.1.6 Impact of COVID-19 on health anxiety in older adults

The COVID-19 pandemic has exacerbated health-related worries among older adults, amplified by information overload. Studies indicate that conflicting health information, particularly misleading content on social media, increased significantly during the pandemic, leading older adults to engage in repetitive searches to verify facts (9). This cycle of information overload and health anxiety may further trigger hypochondriacal behaviors. Additionally, social isolation policies limited older adults’ access to in-person medical consultations, forcing reliance on online platforms. However, due to limited digital literacy, some older adults misinterpreted health information, resulting in unnecessary health fears (10).

#### 4.1.7 Cultural influences on health information search behavior

Health information search behaviors among older adults vary significantly across cultures. For example, Western studies suggest that older adults in Europe and North America rely more on family doctors or professional platforms, whereas Asian older adults (e.g., in China and Japan) are influenced by social media and peer recommendations (11). These differences may shape how health anxiety manifests: Chinese older adults, constrained by “face-saving” cultural norms, often avoid openly discussing health concerns and instead engage in excessive online self-diagnosis, indirectly heightening hypochondriasis risks (12). Future interventions should integrate culturally sensitive approaches.

### 4.2 Interpretation and implications

The results align with cognitive-behavioral theory, suggesting that health-anxious elders are prone to misinterpret online information, leading to compulsive searching and hypochondriasis. Interventions should focus on enhancing digital health literacy and providing reliable health information sources.

### 4.3 Limitations and future directions

1. The sample was limited to one city; future studies should include rural and diverse regional samples.

2. Self-report data may be subject to bias.

3. Digital health literacy was not directly measured; future research should incorporate specific literacy assessments.

## Data Availability

The raw data supporting the conclusions of this article will be made available by the authors, without undue reservation.
